# Relation of *FTO* gene variants to fetal growth trajectories: Findings from the Southampton Women's survey

**DOI:** 10.1016/j.placenta.2015.12.015

**Published:** 2016-02

**Authors:** S.J. Barton, M. Mosquera, J.K. Cleal, A.S. Fuller, S.R. Crozier, C. Cooper, H.M. Inskip, J.W. Holloway, R.M. Lewis, K.M. Godfrey

**Affiliations:** a MRC Lifecourse Epidemiology Unit, Faculty of Medicine, University of Southampton, Southampton SO16 6YD, UK; b Institute of Developmental Sciences, Faculty of Medicine, University of Southampton, Southampton SO16 6YD, UK; c Department of Physiological Sciences, Faculty of Health, University of Valle, Cali, Colombia; d NIHR Musculoskeletal Biomedical Research Unit, University of Oxford, Oxford, OX1 2JD, UK; e Human Genetics and Genomic Medicine, Human Development & Health, Faculty of Medicine, University of Southampton, Southampton SO16 6YD, UK; f NIHR Southampton Biomedical Research Centre, University of Southampton and University Hospital Southampton NHS Foundation Trust, Southampton, SO16 6YD, UK

**Keywords:** Fetal growth trajectories, FTO genotype, MC4R genotype, Placental amino acid transporter expression, Placental FTO expression

## Abstract

**Introduction:**

Placental function is an important determinant of fetal growth, and fetal growth influences obesity risk in childhood and adult life. Here we investigated how FTO and MC4R gene variants linked with obesity relate to patterns of fetal growth and to placental FTO expression.

**Methods:**

Southampton Women's Survey children (n = 1990) with measurements of fetal growth from 11 to 34 weeks gestation were genotyped for common gene variants in FTO (rs9939609, rs1421085) and MC4R (rs17782313). Linear mixed-effect models were used to analyse relations of gene variants with fetal growth.

**Results:**

Fetuses with the rs9939609 A:A FTO genotype had faster biparietal diameter and head circumference growth velocities between 11 and 34 weeks gestation (by 0.012 (95% CI 0.005 to 0.019) and 0.008 (0.002–0.015) standard deviations per week, respectively) compared to fetuses with the T:T FTO genotype; abdominal circumference growth velocity did not differ between genotypes. FTO genotype was not associated with placental FTO expression, but higher placental FTO expression was independently associated with larger fetal size and higher placental ASCT2, EAAT2 and y + LAT2 amino acid transporter expression. Findings were similar for FTO rs1421085, and the MC4R gene variant was associated with the fetal growth velocity of head circumference.

**Discussion:**

FTO gene variants are known to associate with obesity but this is the first time that the risk alleles and placental FTO expression have been linked with fetal growth trajectories. The lack of an association between FTO genotype and placental FTO expression adds to emerging evidence of complex biology underlying the association between FTO genotype and obesity.

## Highlights

• Variants in the *FTO* gene are previously known to be associated with obesity.
• discovered novel associations between *FTO* variants and growth trajectory of fetal head measures.
• also found novel associations between placental *FTO* expression and fetal size.

## 1 Introduction

Obesity is a major public health problem as it increases the risk of coronary heart disease, stroke, metabolic syndrome, some cancers and a wide range of other diseases. Children who are obese are likely to continue to be obese when they become adults, highlighting the need for research into early determinants of obesity [1]. In order to develop effective interventions it is important to understand the genetic and environmental determinants of obesity. There is evidence suggesting critical windows during fetal and early postnatal life, within which altered development may predispose the individual to obesity in later life [2], [3].

Genome wide association studies (GWAS) have been instrumental in the discovery of genes associated with obesity, including the fat mass and obesity-associated (*FTO*) gene [4]. A common variant in the *FTO* gene, rs9939609 has been identified that predisposes to type 2 diabetes through an effect on body mass index (BMI) [4], [5], [6]. *FTO* variants, including rs9939609 and rs1421085, have been reported to be associated with BMI from age 5.5 years [4], [7], [8], [9], [10], [11] and throughout adult life [4], [5], [6], [12], although associations are less consistent in childhood. In addition, one study has shown that *FTO* genotype is associated with fat mass in the first 2 weeks of life [13].

Associations have also been observed between the genotype of a Single Nucleotide Polymorphism (SNP), rs17782313, near the *MC4R* gene [14], [15] and adult obesity. This SNP is also associated with obesity in children [14], [16], [17] and one study reported an association with changes in BMI over the first two weeks of life [18]. *MC4R* genotype is thought to act through an influence on satiety and it is therefore interesting to investigate the effect of this SNP on the fetus where conventional appetite mechanisms cannot play a part.

*FTO* is an RNA demethylase and could act through regulation of mRNA stability [19]. However, no direct connection between obesity associated variants and *FTO* expression and function has been made. A recent study has demonstrated that obesity-associated non-coding variants in *FTO* affect the expression of the gene *IRX3* in humans, mice and zebrafish [20]. Obesity associated SNPs in *FTO* were found to be associated with the expression of *IRX3*, but not *FTO*, in the cerebellum of the human brain. *IRX3* is a homeobox gene involved in pattern formation in the early embryo and is expressed at much lower levels in later life [21]. This raises the possibility that the effects of *FTO* genotype on fetal growth and postnatal obesity may have originated through *IRX3* expression in embryonic life.

Recent research has shown that *FTO* plays a key role in the cellular sensing of amino acids and the regulation of cell growth and global mRNA translation through the mTORC1 pathway [22]. This could explain why carriers of the obesity predisposing SNPs in *FTO* not only consume more calories during test meals but also show an alteration in nutrient preference [23] and a higher dietary protein intake [24]. In utero, fetal growth is regulated by placental amino acid transporters as they control the supply of nutrients to the fetus. Thus *FTO* could potentially regulate fetal growth through a mechanism of altered placental amino acid transport.

The primary aim of this study was to investigate the association of obesity related SNPs (two *FTO* SNPs and one SNP near *MC4R*) with fetal growth throughout gestation in children of the Southampton Women's Survey (SWS) [25]. The secondary aim of this study was to investigate associations between *FTO* genotype, the expression of this gene in the placenta, a fetal tissue, and placental amino acid transporter expression.

## 2 Methods

Offspring of participants in the Southampton Women's Survey were studied [25]. Between 1998 and 2007, 3158 babies were born and data gathered on these babies both during the pregnancy via ultrasound scans and after the birth.

Gestational age of the SWS babies was determined using an algorithm based on last menstrual period, or where this was not available early ultrasound data. Using Acuson 128 XP, Aspen and Sequoia ultrasound machines calibrated to 1540 m/s; experienced research ultrasonographers used standardised anatomical landmarks to measure fetal head, abdominal circumference and biparietal diameter at 11, 19 and 34 weeks gestation.

The method of Royston [26] was used to calculate measures of fetal size, correcting for exact gestational age at measurement.

Maternal smoking during pregnancy was assessed by questionnaire in early and late pregnancy.

DNA was extracted from the cord blood of SWS babies by the salting out method and stored at −80 °C. 1990 children from the SWS cohort with DNA available were genotyped for the following polymorphisms: rs9939609 and rs1421085 located in the *FTO* gene on chromosome 16, and rs17782313 located near the *MC4R* gene on chromosome 18. Genotyping was performed by Kbiosciences (Hoddesdon, Herts, UK) using a custom SNP panel and an in-house calling algorithm. All three SNPs were found to be in HWE (chi squared p value ≥ 0.05); call rate was ≥ 97%; 5% duplicates were included with an error rate of 0%.

We analysed 99 SWS placentas based on availability of neonatal data and Caucasian ethnicity and collection within 30 min of delivery. Five villous tissue samples were selected using a stratified random sampling method and stored at −80 °C and powdered in a frozen tissue press. Total RNA was extracted from 30 mg tissue using the Rneasy fibrous tissue RNA isolation mini kit (Qiagen, UK) according to the manufacturer's instructions. The integrity of total RNA was confirmed by agarose gel electrophoresis. Total RNA (0.2 μg) was reverse transcribed with 0.5 μg random hexamer primer, 200 units M-MLV reverse transcriptase, 25 units recombinant RNasin ribonuclease inhibitor and 0.5 mM each of dATP, dCTP, dGTP and dTTP in a final reaction volume of 25 μl in 1 x MMLV reaction buffer (Promega, Wisconsin, USA). All 99 samples were produced in one batch to reduce variation.

Oligonucleotide probes and primers for *FTO* and the amino acid transporter genes were designed using the Roche (West Sussex, UK) ProbeFinder version 2.45 for human. Probes were supplied by Roche from the human universal probe library and primers were synthesised by Eurogentec (Seraing, Belgium). *FTO* (NM_001080432.2): Forward 5′- cacaacctcggtttagttcca -3′, Reverse 5′- aaatataatccaaggttcctgttgag -3′, probe #53. *ASCT2* (NM_005628.2): Forward 5′-gaggaatatcaccggaacca-3′, Reverse 5′-aggatgttcatcccctcca-3′, probe 43. *EAAT2* (NM_004171.3): Forward 5′-aaaatgctcattctccctctaatc-3′, Reverse 5′-gccactagccttagcatcca-3′, probe 78. *y*^+^*LAT2* (NM_001076785.1): Forward 5′-gctgtgatcccccatacct-3′, Reverse 5′-ggcacagttcacaaatgtcag-3′, probe 66. Control genes were selected using the geNormTM human Housekeeping Gene Selection Kit (Primer Design Limited, Southampton, UK).

*FTO* and amino acid transporter mRNA expression levels were quantified by real-time reverse transcriptase PCR using a Roche LightCycler 480. For Roche universal probe library probes the cycle parameters were 95 °C for 5 min, followed by 45 cycles of 95 °C for 10 s, 60 °C for 30 s and 72 °C for 1 s. For primer design Perfect Probes (control genes) the cycle parameters were 95 °C for 10 min, followed by 40–50 cycles of 95 °C for 15 s, 50 °C for 30 s and 72 °C for 15 s. Intra-assay CV's for each gene were 5–8%. Each of the 99 samples was run on the same plate in triplicate. All mRNA levels are presented relative to the geometric mean of the three control genes, tyrosine 3-monooxygenase/tryptophan 5-monooxygenase activation protein, zeta polypeptide (YWHAZ), ubiquitin C (UBC) and topoisomerase (TOP1) [27].

### 2.1 Statistical analysis

Statistical procedures were performed in Stata version 12 (StataCorp, Texas, USA) and SPSS version 21 (IBM, Armonk, New York). Babies with congenital growth abnormalities and babies of mothers listed as having non-white ethnicity were excluded from analysis.

Anthropometric variables were checked for normality and then standardised to z-scores. For cross-sectional analysis genotypes were coded according to the additive model (0, 1 or 2 copies of the risk allele). The risk allele was considered to be A for *FTO* rs9939609, C for FTO rs1421085 and C for *MC4R* rs17782313. Univariate linear regressions were run with each genotype separately as predictor variable for each outcome (CRL, BPD, HC and AC), adjusting for sex, maternal smoking during pregnancy, pregnancy weight gain category (IOM (2009)) and pre-pregnancy maternal BMI. Results were expressed in SDs of change in outcome per unit increase in number of risk alleles.

**Fig. 1:**
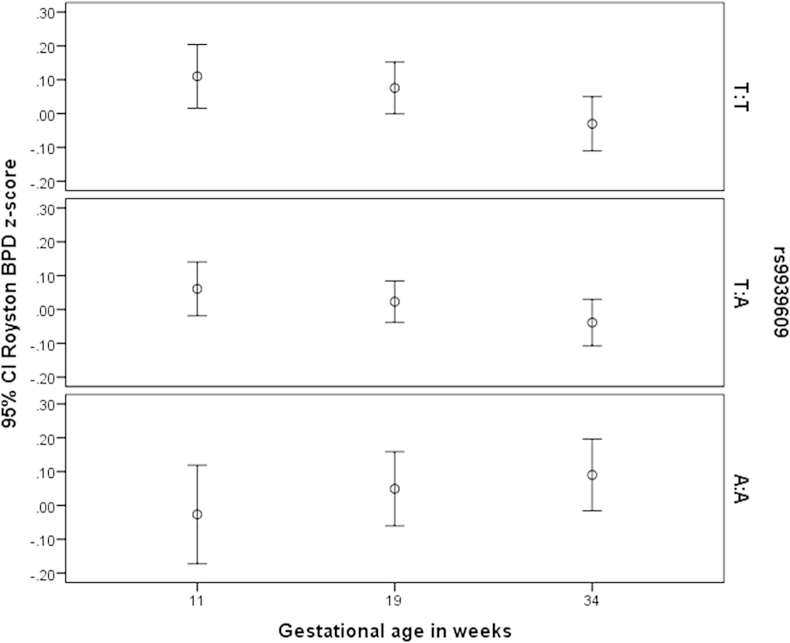
The means and 95% CIs of Royston biparietal diameter z-score at all three available gestational ages for each rs9939609 genotype separately. For the T:T and T:A genotypes the biparietal diameter z-score decreases with increasing gestational age, however for the A:A genotype biparietal diameter z-score increases with gestational age. This shows that the biparietal diameter for the A:A genotype has a significantly different fetal growth trajectory, with fetal biparietal diameter growing faster than the T:T genotype. This was confirmed by linear mixed-effect modelling (p = 0.0009).

Linear mixed-effect models [28] were used to conduct the longitudinal analyses. Intercept and gestational age were entered into the model as random effects and a genotype x gestational age interaction was also included to assess growth velocity in the measurements over time. Sex, maternal smoking, pregnancy weight gain category and pre-pregnancy maternal BMI were included as covariates. Genotype was included as a categorical covariate because exploratory analysis (see Fig. 1) indicated that this was more appropriate.

Partial correlations of *FTO* gene expression in the placenta and anthropometric variables, were calculated, controlling for sex. Neonatal variables were additionally adjusted for gestational age and mode of delivery (Caesarean or vaginal).

Bonferroni correction was applied to account for multiple testing for each of the main hypotheses tested.

## 3 Results

Analysis of fetal growth, was based on 1990 SWS singleton births with available fetal measurements and *FTO* or *MC4R* genotype or placental gene RNA data. Supplementary Figure 1 shows the numbers of SWS babies with available DNA and genotype. Table 1 shows summary statistics for the SWS cohort divided into groups according to whether genotype was available or not. There does not appear to be much difference between these groups.

The minor allele frequency for the *FTO* rs9939609 genotype in the SWS was A = 0.41, similar to the minor allele frequency quoted in HapMap [29] for Caucasians (CEU: A = 0.45).

### 3.1 Cross-sectional analysis

Table 2 shows there were a number of potentially significant associations at the 5% level between *FTO* and *MC4R* SNPs and fetal anthropometric measurements. While none of these associations remained significant at p < 0.05 after a strict Bonferroni correction, many of the fetal measures are correlated, so a Bonferroni correction is likely to be overly conservative. A retrospective power calculation indicated that to find a difference in means of fetal measure z-scores between genotype groups of 0.18, 1446 SWS children would be required to achieve a power of 90% at a 5% significance level.

Fig. 1 shows the means and 95% confidence intervals for biparietal diameter z-score at 11, 19 and 34 weeks for the *FTO* rs9939609 genotypes. At 11 weeks gestation, fetuses with the A:A genotype tended to have a lower biparietal diameter z-score than either the T:A genotype or the T:T genotype. At 19 weeks, biparietal diameter z-score was similar in the three genotypes. However, at 34 weeks gestation, fetuses with the A:A genotype had a higher biparietal diameter z-score than either of the other two genotypes. This implies a different fetal biparietal diameter growth trajectory in fetuses with the A:A genotype compared to the other two genotypes, with a smaller 11 week size followed by a faster growth velocity for the A:A genotype.

### 3.2 Linear mixed-effect models

Maternal weight gain category and sex were significantly associated with biparietal diameter, head circumference and abdominal circumference when included in linear mixed-effect models (p < 0.0001), with males having a larger measurement than females. Maternal smoking in pregnancy was associated with biparietal diameter and head circumference (p ≤ 0.05), with the fetuses of mothers who did not smoke in pregnancy having a larger head size. Maternal smoking in pregnancy was not associated with abdominal circumference (p > 0.05). Maternal pre-pregnancy BMI was significantly associated with head circumference and abdominal circumference (p ≤ 0.007) but not with biparietal diameter.

Linear mixed-effect models for two measures of head size, namely biparietal diameter and head circumference z-score, measured at 11, 19 and 34 weeks gestation, showed that *FTO* genotype rs9939609 was strongly associated with the growth velocity of head size, as there was a significant interaction between genotype and gestational age (p = 0.0009 for biparietal diameter and p = 0.014 for head circumference). This demonstrates that *FTO* genotypes have different growth trajectories. When abdominal circumference z-score was used as the dependent variable, there was no association between the growth velocity of abdominal circumference and *FTO* genotype. The results from linear mixed-effect models analysing the rs1421085 genotype were very similar to those using the rs9939609 genotype due to the high LD between them (data not shown). When the *MC4R* genotype rs17782313 was used as a predictor in the longitudinal models there were no significant associations between this SNP and the growth velocity of biparietal diameter and abdominal circumference, but there was an association between *MC4R* and head circumference growth velocity (p = 0.013) across the three gestational ages of 11, 19 and 34 weeks.

Interactions between *FTO* genotype and sex, and between *FTO* genotype and maternal smoking were investigated but no significant interactions were found (p > 0.05).

### 3.3 Placental *FTO* relative RNA level

Placental *FTO* relative RNA level was available for 99 offspring (mean 1.23, sd 0.27).

**Fig. 2:**
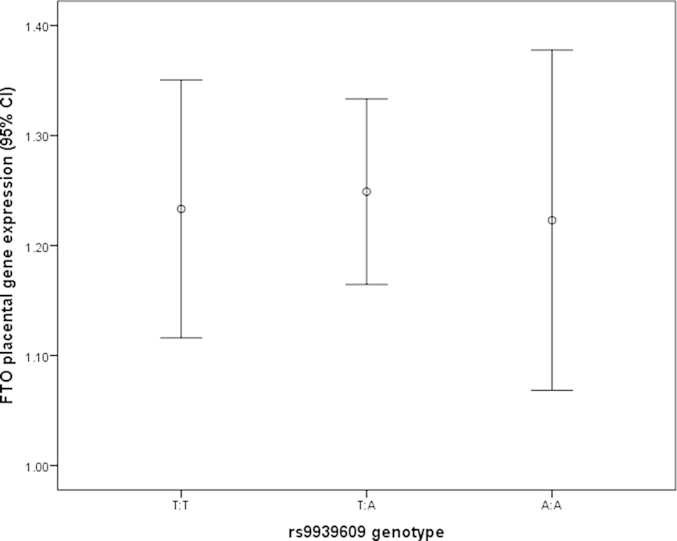
There is no association between *FTO* genotype and expression of *FTO* in the placenta (ANOVA p value = 0.914).

No association was observed between *FTO* genotype and relative RNA levels of *FTO* in the placenta (assessed by ANOVA, p value = 0.914, Fig. 2).

**Fig. 3:**
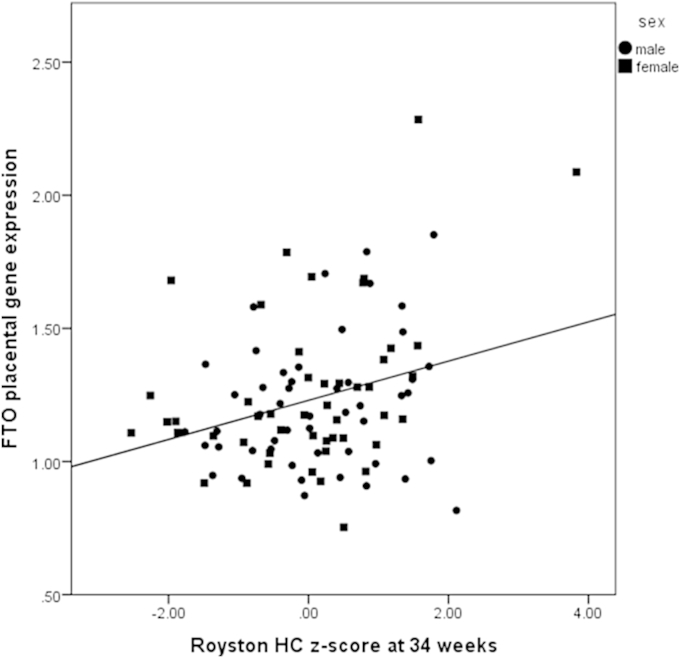
The correlation between *FTO* relative RNA level in the placenta and 34 week Royston head circumference z-score (r = 0.324, p = 0.001, n = 98). Males were plotted using circles and females using squares.

Table 3 shows partial correlations, controlling for sex, of fetal anthropometric measurements with placental *FTO* relative gene expression. Neonatal measures were adjusted for sex, gestational age and mode of delivery. Several fetal anthropometric measurements appeared highly correlated with the *FTO* expression in the placenta (Table 3, Fig. 3). Head circumference at 34 weeks was significantly associated with placental *FTO* relative gene expression after Bonferroni correction and neonatal head circumference showed a borderline association.

We also attempted to measure the levels of *IRX3* in the term human placenta, but found that levels were too low to measure reliably.

Associations were also found between *FTO* mRNA expression and the expression of placental amino acid transporters. *FTO* expression was correlated with *ASCT2* (rho = 0.29, p = 0.004), *EAAT2* (rho = 0.22, p = 0.027) and *y*^+^*LAT2* (rho = 0.37, p = 0.0002) mRNA expression.

## 4 Discussion

We have shown novel associations between *FTO* genotypes rs9939609 and rs1421085 and two measures of fetal head growth. From 11 to 34 weeks gestation fetuses with the homozygous *FTO* risk genotype (A:A or C:C) showed a higher velocity of biparietal diameter and head circumference growth in comparison with the other genotypes (T:A or T:C and T:T). Results were also in accordance with previous publications showing that maternal smoking is significantly associated with smaller fetal head size [30]. Trends were also identified in our cross-sectional analyses between the *FTO* SNPs and a SNP near *MC4R* and head circumference and crown-rump length, although these did not survive Bonferroni correction. For the above associations, the effect was in accordance with published literature for adults and babies [14], [15], [18] as the addition of each risk allele increased the measurements. We did not observe associations between the *MC4R* SNP and fetal growth trajectories of biparietal diameter and abdominal circumference, or between *FTO* SNPs and the fetal abdominal circumference growth trajectory.

Few authors have investigated the possible effect of *FTO* SNPs on fetal growth. Marsh et al. [31] reported that there was no evidence of any difference in fetal growth between the sexes and between rs9930609 genotype up to 28 weeks gestation. After 28 weeks gestation they observed a significant association between the rs9939609 A:A genotype and fetal growth restriction in non-smoking Australian mothers and fetal growth enhancement for mothers who smoked. This differs from our results as we did not observe a significant interaction between maternal smoking and genotype. However, the two sets of results are difficult to compare as our measurements were made by research staff following strict measurement protocols and we have considered the growth trajectory over all available gestational ages in the Southampton Women's Survey cohort; in contrast Marsh et al. used routinely collected data and analysed each trimester of pregnancy separately.

Placental function is an important determinant of fetal growth and may predispose to obesity later in life [32]. Our observations that *FTO* gene expression was correlated with birth weight are consistent with those of previous studies [33], [34], [35]. In addition we found novel associations between *FTO* gene expression and fetal growth parameters. However, placental *FTO* expression was not associated with either of the *FTO* genotypes in our SWS cohort, consistent with a previous report [34]. It is now thought that mutations in Intron 1 of the *FTO* gene control the expression of *IRX3* [20]. Our observation that FTO expression was independent of its genotype is consistent with the suggestion that the associations between FTO genotype and fetal growth are not mediated via FTO expression. If *FTO* is not regulated by genotype the question arises as to what is regulating *FTO* expression. *FTO* may be involved in nutrient sensing and regulate the mTOR pathway and, in the placenta, *FTO* gene expression may be related to maternal nutritional status. We observed that FTO gene expression was related to the expression of specific amino acid transporters in the placenta. As amino acid transfer is essential for fetal development this provides a potential mechanism by which FTO expression within the placenta may affect placental function and therefore fetal growth [36].

Consistent with the observation that *FTO* genotype is associated with fetal head growth; in humans a ‘loss of function’ mutation in the *FTO* gene has been found to induce postnatal growth retardation and malformations in the head and brain [37] Other recent studies have reported that carriers of common *FTO* gene polymorphisms show both a reduction in frontal lobe volume of the brain [38] and an impaired verbal fluency performance [39], It was interesting that *FTO* genotype (thought to be controlling IRX3 gene expression) and *FTO* gene expression (which is independent of *FTO* genotype rs9939609) were both associated with fetal head growth in our study, however it is not clear whether there is a biological relationship underlying this. *FTO* knockout mice have altered head growth [40], but as knocking out the *FTO* gene also knocks out the *FTO* SNPs regulating *IRX3* it is not clear whether the effects of *FTO* knockout is due to the loss of *FTO* gene expression or the loss of regulation of the *IRX3* gene.

Fetal growth is a complex trait which is likely to be determined by multiple genetic [41] and environmental [42] factors. The cumulative effect of multiple variants could produce clinically relevant differences in growth. The detection of novel genetic variants associated with fetal growth has the potential to identify molecular mechanisms connected with growth and can yield insights of biological importance.

## Author contributions

SJB contributed to the design of the study, performed the statistical analysis and interpretation of the results and drafted the article. SRC contributed to the statistical analysis, acquisition of data and interpretation of the results. MM contributed to acquisition of data, analysis and interpretation of data. JKC contributed to the design and interpretation of data. ASF contributed to the statistical analysis and interpretation of the data. CC contributed to the design, acquisition and interpretation of data. HMI contributed to acquisition of data, interpretation of data, revision and final approval of paper. JWH contributed to design, acquisition of data and interpretation of the data. RML contributed to sample collection, design and interpretation of the data. KMG contributed to design, analysis and interpretation of data. All authors reviewed and approved the final version of the article.

## Conflict of interest

Keith Godfrey has received reimbursement for speaking at conferences sponsored by companies selling nutritional and pharmaceutical products. One of the research groups involved in this work are part of an academic consortium that has received funding from Abbott Nutrition, Nestec and Danone.

## Ethical approval

The study was conducted according to the guidelines in the Declaration of Helsinki, and the Southampton and South West Hampshire Research Ethics Committee approved all procedures (276/97, 307/97).

## Informed consent

Written informed consent was obtained from all participating women and by parents or guardians with parental responsibility on behalf of their children.

## Figures and Tables

**Table 1:** Summary statistics for the Southampton Women’s Survey Cohort, showing subgroups defined by availability of genotype

Variable	Genotype available			Genotype not available		
N	Mean	Std. Dev.	N	Mean	Std. Dev.
11 week scan: Crown rump length (mm)	1593	53.48	9.07	808	53.32	8.88
11 week scan: Biparietal diameter (mm)	1247	18.72	2.59	609	18.66	2.52
19 week scan: Biparietal diameter (mm)	1912	45.5	2.54	961	45.75	2.49
34 week scan: Biparietal diameter (mm)	1869	86.98	3.6	896	87.09	3.51
11 week scan: Head circumference (mm)	1223	70.46	9.41	602	69.99	8.94
19 week scan: Head circumference (mm)	1912	168.43	8.53	960	168.79	8.53
34 week scan: Head circumference (mm)	1869	317.94	10.98	902	317.42	11.02
Birth: Head circumference (mm)	1955	350.33	13.47	848	348.74	14.87
11 week scan: Abdominal circumference (mm)	1152	55.99	7.91	555	55.96	7.42
19 week scan: Abdominal circumference (mm)	1902	146.46	8.95	960	146.97	8.75
34 week scan: Abdominal circumference (mm)	1958	307.87	15.42	940	307.22	15.02
Birth: Abdominal circumference (mm)	1953	318	20.18	846	315.22	21.86
Birth: Birthweight (g)	1976	3485.49	506.91	984	3373.68	623.77
Maternal pre-pregnancy BMI (kg/m2)	1964	25.35	4.77	999	25	4.78
Sex	1982	51.6% male		1008	52.5% male	
IOM maternal weight gain	1744	28.5% adequate		600	31.7% adequate	
maternal smoking	1944	16.9% yes		887	16.1% yes	
maternal pre-eclampsia	1982	2.8% yes		1008	3.3% yes	
maternal gestational diabetes	1982	1.1% yes		1008	1.0% yes	
SGA (WHO UK z-score )	1976	7.5% yes		984	8.8% yes	

**Table 2:** Regression results for cross-sectional analysis of fetal measurements, controlling for sex, maternal smoking during pregnancy, gestational weight gain and maternal BMI. Beta values represent change in fetal measure per risk allele, p values are uncorrected.

Variable	FTO rs9939609			FTO rs1421085			MC4R rs17782313		
beta	se	p value	beta	se	p value	beta	se	p value
11 week scan: Royston CRL z-score	-0.066	0.04	0.099	-0.081	0.039	0.04	0.088	0.046	0.056
11 week scan: Royston BPD z-score	-0.074	0.043	0.083	-0.076	0.042	0.073	-0.026	0.049	0.592
19 week scan: Royston BPD z-score	-0.023	0.032	0.472	-0.033	0.032	0.299	0.025	0.038	0.509
34 week scan: Royston BPD z-score	0.034	0.035	0.33	0.022	0.034	0.526	0.05	0.04	0.211
11 week scan: Royston HC z-score	-0.11	0.042	0.013	-0.1	0.042	0.016	-0.061	0.049	0.22
19 week scan: Royston HC z-score	-0.031	0.032	0.332	-0.035	0.031	0.266	0.03	0.037	0.41
34 week scan: Royston HC z-score	-0.011	0.034	0.74	-0.016	0.034	0.644	0.059	0.04	0.136
11 week scan: Royston AC z-score	-0.071	0.047	0.128	-0.083	0.047	0.076	-0.048	0.053	0.373
19 week scan: Royston AC z-score	-0.027	0.032	0.396	-0.03	0.031	0.332	0.047	0.036	0.201
34 week scan: Royston AC z-score	-0.022	0.033	0.51	-0.025	0.033	0.446	0.054	0.039	0.16
Birth weight z-score (also controlling for GA)	0.004	0.03	0.888	0.012	0.03	0.678	0.052	0.035	0.142

**Table 3:** Partial correlations, controlling for sex, of anthropometric measurements and placental *FTO* relative RNA level

Variable	Correlation	Significance (2-tailed)	N
11 week scan: Royston CRL z-score	0.289	0.020	66
11 week scan: Royston BPD z-score	0.359	0.014	47
19 week scan: Royston BPD z-score	0.212	0.045	91
34 week scan: Royston BPD z-score	0.241	0.018	97
11 week scan: Royston HC z-score	0.205	0.172	47
19 week scan: Royston HC z-score	0.180	0.090	91
34 week scan: Royston HC z-score	0.324	0.001	98
11 week scan: Royston AC z-score	0.345	0.023	44
19 week scan: Royston AC z-score	0.179	0.091	91
34 week scan: Royston AC z-score	0.242	0.017	98
Birth weight (controlling for sex, GA and mode of delivery)	0.204	0.046	99
Neonatal HC (controlling for sex, GA and mode of delivery)	0.276	0.006	99
Neonatal AC (controlling for sex, GA and mode of delivery)	0.165	0.107	99
