# Supplementary material for: Relation of FTO gene variants to fetal growth trajectories: Findings from the Southampton Women's survey
Source: Placenta. 2016 Feb;38:100–6. doi: 10.1016/j.placenta.2015.12.015 (PMC4776702; doi:10.1016/j.placenta.2015.12.015)
Supplement: Supplementary file 1 [file mmc1.pptx]

## Slide 1
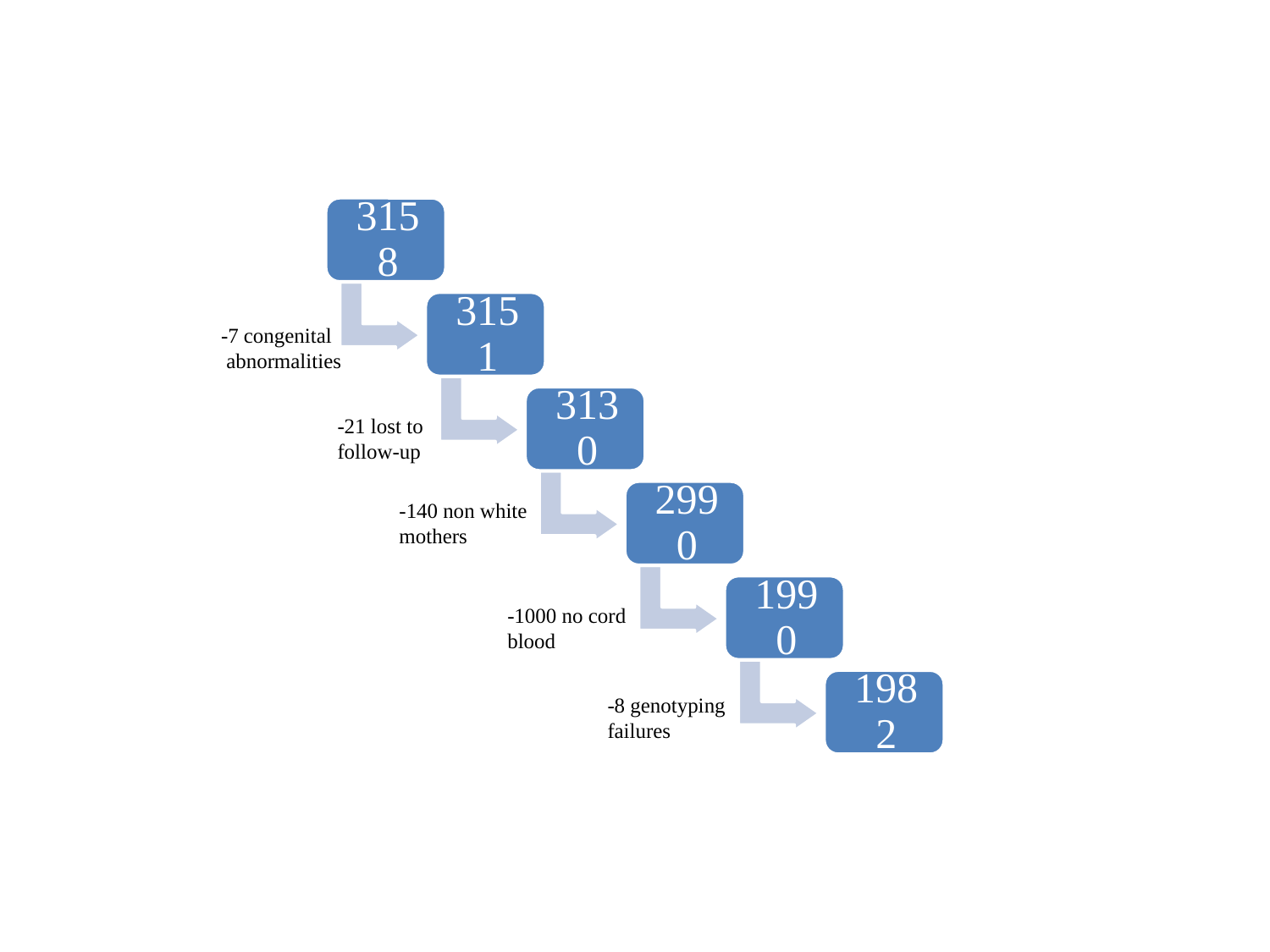

-7 congenital
 abnormalities
-21 lost to follow-up
-140 non white mothers
-1000 no cord blood
-8 genotyping failures
